# Tacrolimus Ophthalmic Suspension Can Be an Effective Treatment Option for Biologic-Induced Refractory Conjunctivitis

**DOI:** 10.7759/cureus.85373

**Published:** 2025-06-04

**Authors:** Yoshihito Mima, Masako Yamamoto, Ken Iozumi

**Affiliations:** 1 Department of Dermatology, Tokyo Metropolitan Police Hospital, Tokyo, JPN

**Keywords:** atopic dermatitis, conjunctivitis, interleukin-4/13, lebrikizumab, tacrolimus eye drops

## Abstract

Atopic dermatitis (AD) is a chronic, relapsing inflammatory skin disease characterized by intense pruritus. It is a multifactorial condition involving complex interactions among skin barrier dysfunction, immune dysregulation, genetic predisposition, and alterations in the skin microbiome. The disease is primarily driven by Th2-associated cytokines such as interleukin (IL)-4, IL-13, and IL-31, which contribute to inflammation and exacerbate pruritus, perpetuating the "itch-scratch cycle." Recently, biologics targeting Th2 cytokines - such as dupilumab, lebrikizumab, and tralokinumab - have emerged as effective treatment options for moderate-to-severe AD. Conjunctivitis is a common adverse effect associated with biologic therapies. In severe cases of conjunctivitis, continuation of biologic therapy may be difficult, highlighting the importance of appropriate ophthalmologic management. Herein, we report a case of AD successfully controlled with lebrikizumab, in which the patient developed conjunctivitis refractory to artificial tears and fluorometholone eye drops. Switching to tacrolimus ophthalmic suspension resulted in marked improvement. While tacrolimus eye drops are not approved for conjunctivitis, they are indicated for vernal keratoconjunctivitis and may be considered in severe cases of AD-related conjunctivitis in consultation with ophthalmologists.

## Introduction

Atopic dermatitis (AD) is a chronic, relapsing, non-infectious inflammatory skin disease characterized primarily by persistent pruritus. Clinically, it presents as eczematous lesions such as erythema, papules, and exudation, with lesion distribution and morphology varying by age. Chronic inflammation is often exacerbated by scratching, leading to lichenification and skin thickening. Intense itching interferes with daily life and sleep, significantly impairing quality of life (QOL) [1,2]. It is a multifactorial disease involving genetic predisposition, skin barrier dysfunction, immune dysregulation, alterations in the skin microbiome, and environmental allergens [3]. A family history is strongly correlated with disease onset, and loss-of-function mutations in the filaggrin (FLG) gene are recognized as major risk factors [4]. Immunologically, AD is predominantly driven by Th2-mediated inflammation, with overproduction of cytokines such as IL-4, IL-13, IL-5, and IL-31. Recent studies also implicate Th1, Th17, and Th22 pathways, with immune profiles varying by age and ethnicity [5-8]. Notably, IL-13 and IL-31 promote the growth of sensory nerves, thereby intensifying pruritus [9]. This contributes to the formation of an “itch-scratch cycle,” wherein inflammatory cytokines induce itch, and scratching further aggravates inflammation [10]. Dysbiosis of the skin microbiota also plays a role, particularly the overgrowth of Staphylococcus aureus, which impairs barrier function and promotes inflammation [11]. Thus, AD is a complex disorder involving genetic, barrier, immune, microbial, and environmental factors [1-11].

In recent years, biologic therapies targeting Th2 cytokines, such as dupilumab, lebrikizumab, and tralokinumab, have emerged as promising options for moderate-to-severe AD unresponsive to topical treatments [12]. These agents significantly improve pruritus and eczema; however, conjunctivitis has been reported as an adverse event in approximately 10% of patients receiving these biologics, with higher incidence in more severe cases [13]. Conjunctivitis, an inflammation or infection of the conjunctiva, can result from allergens, irritants, bacteria, or viruses, and manifests with symptoms such as redness, itching, burning, discharge, and eyelid edema. Diagnosis of immunomodulator-associated conjunctivitis typically requires ophthalmologic evaluation to exclude other causes, using thorough history-taking, clinical assessment, and slit-lamp examination. Treatment usually involves artificial tears, antihistamine drops (e.g., olopatadine), and topical corticosteroids (e.g., fluorometholone), but many cases remain refractory and difficult to manage [14]. In severe cases, biologic therapy may need to be discontinued, making appropriate management of conjunctivitis a critical issue in AD treatment [11-14].

Here, we report a case of a patient with AD who developed lebrikizumab-induced conjunctivitis. Despite two months of treatment with artificial tears, olopatadine, and fluorometholone eye drops, the symptoms persisted. However, switching to tacrolimus ophthalmic suspension resulted in marked improvement, highlighting its potential utility in managing biologic-induced conjunctivitis.

## Case presentation

**Figure 1:**
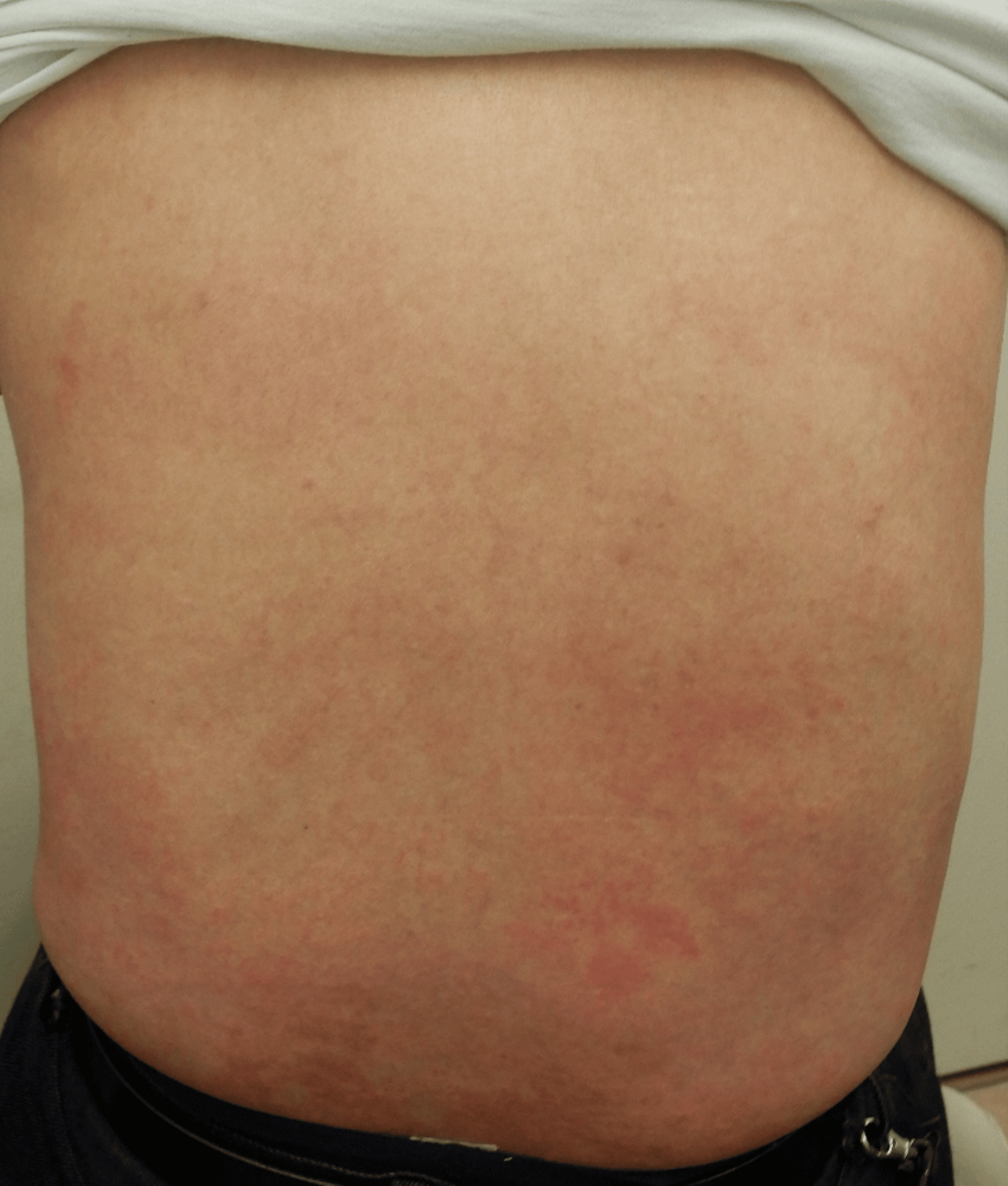
Physical examination revealed extensive erythema and eczematous plaques across the entire back, accompanied by intense pruritus.

A 50-year-old man with a longstanding history of atopic dermatitis since childhood had been treated with topical corticosteroids such as betamethasone butyrate propionate for many years. However, his pruritus and eczematous lesions persisted and remained refractory. He was referred to our clinic for escalation of treatment. On initial examination, widespread eczematous lesions were observed on the trunk and extremities (Figure 1).

The Eczema Area and Severity Index (EASI) score was 21.2, the Investigator’s Global Assessment (IGA) score was 4, and the Peak Pruritus Numerical Rating Scale (PP-NRS) score was 9, indicating severe pruritus that significantly impaired the patient’s sleep. Laboratory findings are summarized in Table 1. Elevated levels of lactate dehydrogenase (LDH), immunoglobulin E (IgE), and thymus and activation-regulated chemokine (TARC) were consistent with active inflammation and pronounced allergic activity associated with atopic dermatitis.

**Table 1:** The results of laboratory examination at the first visit RR = Reference range; AST = Aspartate aminotransferase; ALT = Alanine aminotransferase; LDH = Lactate dehydrogenase; CRP = C-reactive protein; IgG = Immunoglobulin G; IgA = Immunoglobulin A; IgM = Immunoglobulin M; IgE = Immunoglobulin E; TARC = Thymus and activation-regulated chemokine

Variable	Patient value	RR, adults
AST	16 U/L	11–33 U/L
ALT	19 U/L	6–37 U/L
Albumin	4.2 g/dL	3.8–5.0 g/dL
Total protein	6.6 g/dL	6.1–8.2 g/dL
Total bilirubin	0.7 mg/dL	0.2–1.2 mg/dL
LDH	321 U/L	135–214 U/L
CRP	0 mg/dL	< 0.50 mg/dL
Creatinin	0.70 mg/dl	0.6–1.1 mg/dl
IgG	832 mg/dL	870–1700 mg/dL
IgA	323 mg/dL	110–410 mg/dL
IgM	31 mg/dL	35–220 mg/dL
Eosinophil	1000 /μl	0-500 /μl
IgE	9760 IU/ml	0-170 IU/ml
TARC	1395 pg/ml	0-450 pg/ml

**Figure 2:**
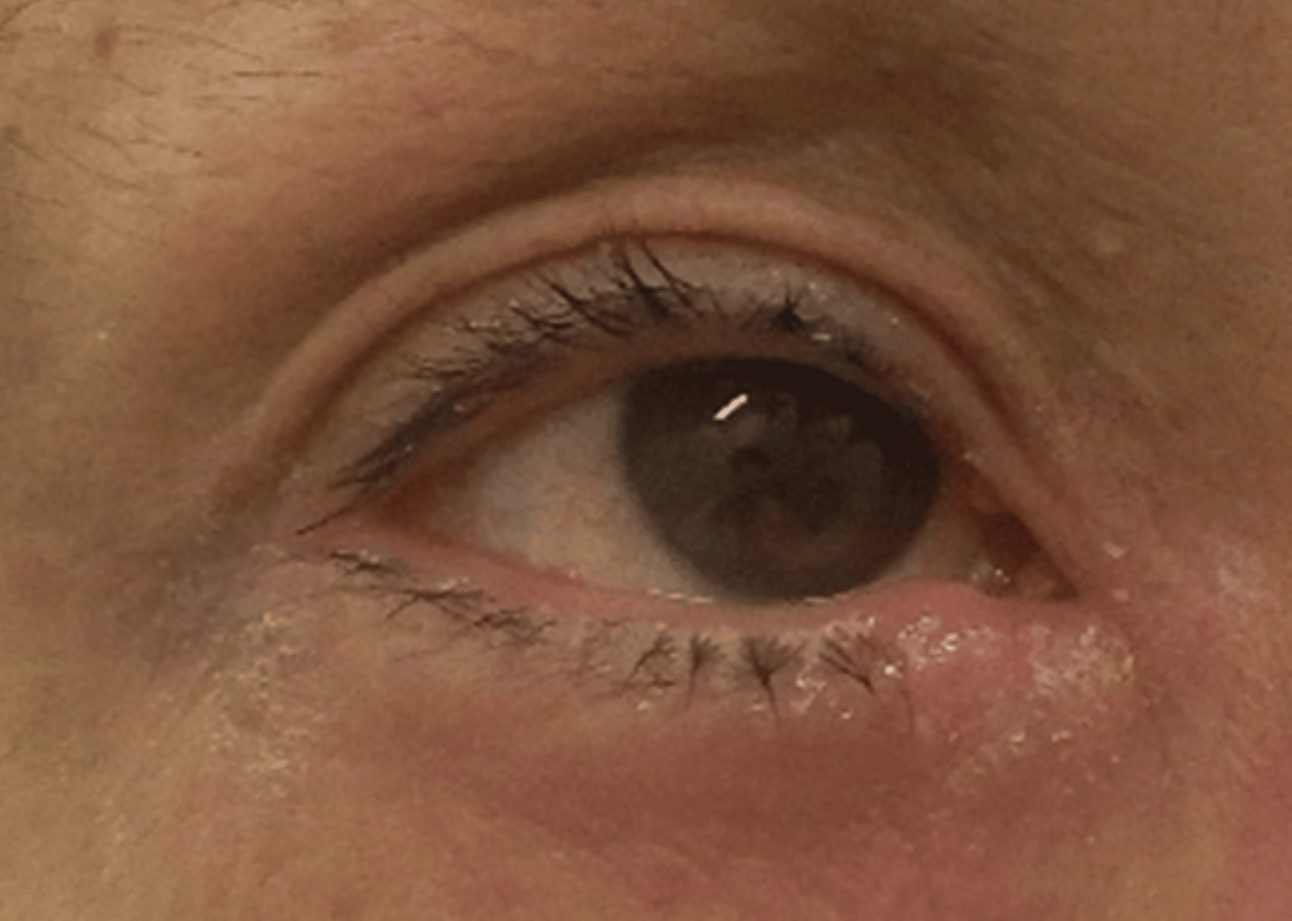
Marked conjunctival injection and pronounced erythema around the eyelids were observed.

Various treatment options were considered, including oral corticosteroids, oral cyclosporine, injectable biologics, and oral Janus kinase (JAK) inhibitors. The patient preferred upadacitinib due to its oral administration, suitability for long-term use, and rapid antipruritic effect. Given concerns about potential side effects such as herpes zoster, treatment was initiated with upadacitinib at a dose of 15 mg/day. Within two weeks, his pruritus improved to a PP-NRS score of 4, and his skin lesions also began to show improvement. After four months of continued upadacitinib therapy, the skin condition had markedly improved; however, the patient continued to experience fluctuating pruritus throughout the day. At his request, the treatment was switched to lebrikizumab, administered as 500 mg for the first and second doses, followed by 250 mg every two weeks. Two months after the fourth injection, both pruritus and skin symptoms were well controlled, with the PP-NRS score improving to 1. However, approximately one month after initiating lebrikizumab, the patient developed ocular pruritus and conjunctival injection (Figure 2). Despite two months of treatment with olopatadine and fluorometholone eye drops prescribed by a local ophthalmologist, there was no improvement.

**Figure 3:**
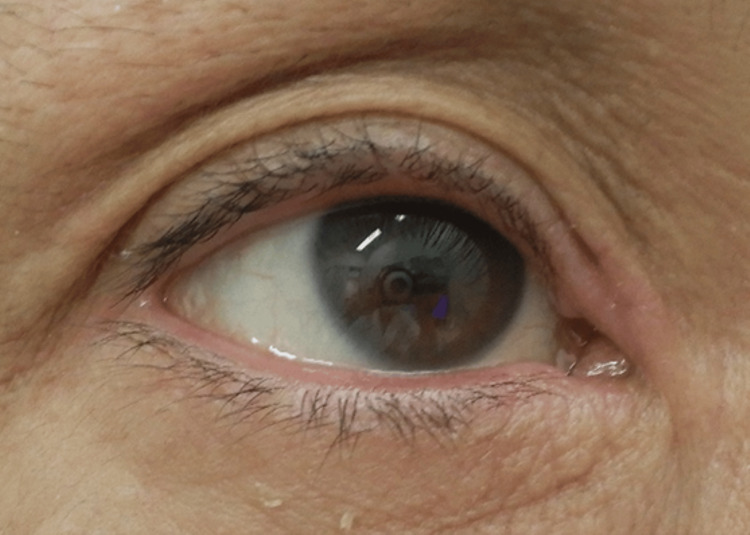
Conjunctival injection and periocular erythema completely resolved, and the pruritus also subsided.

We discussed the option of switching to an alternative biologic agent such as tralokinumab; however, the patient preferred to continue lebrikizumab, as his systemic pruritus and skin symptoms were well controlled. As the conjunctivitis worsened, prominent papillary hypertrophy of the palpebral conjunctiva developed, resembling vernal keratoconjunctivitis. The treatment was switched to tacrolimus ophthalmic suspension (Talymus), which resulted in complete resolution of ocular pruritus and conjunctival injection within three weeks (Figure 3). Currently, the patient continues lebrikizumab while using Talymus eye drops, and both pruritus and skin symptoms associated with atopic dermatitis remain well controlled.

## Discussion

The exact mechanisms underlying conjunctivitis induced by biologics such as dupilumab and lebrikizumab remain unclear. One proposed hypothesis is that inhibition of IL-4 and IL-13 signaling may lead to upregulation of ligands such as OX40L, contributing to the development of atopic keratoconjunctivitis. Additionally, both agents are known to cause transient eosinophilia, and given the role of eosinophils in allergic ocular diseases, this may theoretically increase the risk of conjunctivitis [15]. Recent reports have also suggested that impaired tear secretion may be associated with conjunctivitis in some patients with AD treated with dupilumab. Enzymes and proteins produced by the lacrimal glands play roles in chemical signaling and immune defense, and IL-4/IL-13 inhibition may downregulate the genes encoding these molecules, leading to tear film dysfunction and conjunctival inflammation [16].

First-line treatment for conjunctivitis includes artificial tears, antihistamine eye drops such as olopatadine, and topical corticosteroids such as fluorometholone 0.1%, which suppress both inflammation and allergic responses. Fluorometholone is considered effective for biologic-induced conjunctivitis and has a favorable safety profile with lower risk of cataract and glaucoma compared to other corticosteroids, although prolonged use still requires caution. Although dermatologists may be less familiar with their use, immunosuppressive eye drops such as cyclosporine and tacrolimus can be considered in refractory cases. Cyclosporine eye drops are not approved for conjunctivitis and may cause local irritation, burning, or pain. Tacrolimus eye drops are also off-label for conjunctivitis, but clinical studies have demonstrated efficacy, with the added benefit of not increasing the risk of cataract or glaucoma with long-term use. In fact, several cases of dupilumab-associated conjunctivitis unresponsive to corticosteroids have shown marked improvement with a switch to cyclosporine or tacrolimus eye drops [17,18].

In our case, the patient was referred to a local ophthalmologist after developing severe allergic conjunctivitis with giant papillary hypertrophy of the palpebral conjunctiva, raising suspicion of vernal keratoconjunctivitis. Switching from fluorometholone to tacrolimus ophthalmic suspension led to marked improvement in lebrikizumab-induced conjunctivitis. In daily practice, it is not uncommon for severe conjunctivitis to necessitate discontinuation of biologic therapy. While JAK inhibitors such as upadacitinib have a slightly higher incidence of systemic side effects, including hematologic and hepatic abnormalities, the incidence of conjunctivitis is substantially lower (approximately 1.5%) compared to biologics [19,20]. Moreover, in patients with AD and concomitant vernal keratoconjunctivitis-like symptoms, treatment with upadacitinib has been reported to improve both skin and ocular symptoms [19,20]. In the present case, switching to a JAK inhibitor such as abrocitinib was considered if ocular symptoms persisted, but since Talymus was highly effective, lebrikizumab therapy was continued. While cyclosporine and tacrolimus eye drops are not approved for conjunctivitis, they are indicated for vernal keratoconjunctivitis in Japan, and should be considered in collaboration with ophthalmologists for patients receiving biologics who develop significant ocular symptoms.

Although lebrikizumab is a newly approved treatment, it may cause conjunctivitis similar to dupilumab [20]. Our case suggests that tacrolimus eye drops could be a potential treatment option, as with dupilumab-associated conjunctivitis. Effective control of ocular symptoms through topical immunosuppressants may allow continuation of biologic therapy including lebrikizumab, contributing to more optimal treatment strategies for AD. Therefore, we should remain aware of these therapeutic options and coordinate care with ophthalmologists when necessary. Further clinical studies are needed to evaluate the effectiveness of these treatments in managing immunomodulator-induced conjunctivitis.

## Conclusions

The pathogenesis of conjunctivitis associated with dupilumab and lebrikizumab is thought to involve several factors, including upregulation of OX40L activity, transient eosinophilia, and downregulation of genes related to tear production. However, the precise mechanisms remain poorly understood. In dermatologic practice, artificial tears, antihistamine eye drops, and topical corticosteroids such as fluorometholone are commonly used, but a subset of patients presents with refractory conjunctivitis that does not respond adequately to these therapies. As demonstrated in our case, switching to tacrolimus ophthalmic suspension may offer substantial benefit in such instances. If ocular symptoms can be effectively controlled with topical therapy, continuation of biologic treatment becomes feasible, enabling a more optimal and sustained management strategy for atopic dermatitis. This case underscores the importance of managing ocular complications to maintain effective biologic therapy and provides insight into future treatment strategies for atopic dermatitis. Moving forward, further clinical evidence is needed to better understand the efficacy of immunosuppressive eye drops such as tacrolimus and cyclosporine in managing biologic-associated conjunctivitis.

## References

[1] Asher MI, Montefort S, Björkstén B, Lai CK, Strachan DP, Weiland SK, Williams H (2006). ISAAC Phase Three Study Group. Worldwide time trends in the prevalence of symptoms of asthma, allergic rhinoconjunctivitis, and eczema in childhood: ISAAC Phases One and Three repeat multicountry cross-sectional surveys. Lancet.

[2] Avena-Woods C (2017). Overview of atopic dermatitis. Am J Manag Care.

[3] Sroka-Tomaszewska J, Trzeciak M (2021). Molecular mechanisms of atopic dermatitis pathogenesis. Int J Mol Sci.

[4] Irvine AD, McLean WH, Leung DY (2011). Filaggrin mutations associated with skin and allergic diseases. N Engl J Med.

[5] Cosmi L, Maggi L, Mazzoni A, Liotta F, Annunziato F (2019). Biologicals targeting type 2 immunity: lessons learned from asthma, chronic urticaria and atopic dermatitis. Eur J Immunol.

[6] Matsunaga MC, Yamauchi PS (2016). IL-4 and IL-13 inhibition in atopic dermatitis. J Drugs Dermatol.

[7] Brunner PM, Guttman-Yassky E, Leung DY (2017). The immunology of atopic dermatitis and its reversibility with broad-spectrum and targeted therapies. J Allergy Clin Immunol.

[8] Czarnowicki T, He H, Krueger JG, Guttman-Yassky E (2019). Atopic dermatitis endotypes and implications for targeted therapeutics. J Allergy Clin Immunol.

[9] Meng J, Moriyama M, Feld M (2018). New mechanism underlying IL-31-induced atopic dermatitis. J Allergy Clin Immunol.

[10] Yosipovitch G, Rosen JD, Hashimoto T (2018). Itch: from mechanism to (novel) therapeutic approaches. J Allergy Clin Immunol.

[11] Alexander H, Paller AS, Traidl-Hoffmann C (2020). The role of bacterial skin infections in atopic dermatitis: expert statement and review from the International Eczema Council Skin Infection Group. Br J Dermatol.

[12] Salvati L, Cosmi L, Annunziato F (2021). From emollients to biologicals: targeting atopic dermatitis. Int J Mol Sci.

[13] Aszodi N, Thurau S, Seegräber M, de Bruin-Weller M, Wollenberg A (2019). Management of dupilumab-associated conjunctivitis in atopic dermatitis. J Dtsch Dermatol Ges.

[14] Wollenberg A, Ariens L, Thurau S, van Luijk C, Seegräber M, de Bruin-Weller M (2018). Conjunctivitis occurring in atopic dermatitis patients treated with dupilumab-clinical characteristics and treatment. J Allergy Clin Immunol Pract.

[15] Mennini M, Dahdah L, Fiocchi A (2017). Two phase 3 trials of dupilumab versus placebo in atopic dermatitis. N Engl J Med.

[16] Han H, Cummings S, Shade KC (2023). Cellular mechanisms and effects of IL-4 receptor blockade in experimental conjunctivitis evoked by skin inflammation. JCI Insight.

[17] Fukushima A, Ohashi Y, Ebihara N (2014). Therapeutic effects of 0.1% tacrolimus eye drops for refractory allergic ocular diseases with proliferative lesion or corneal involvement. Br J Ophthalmol.

[18] Bohner A, Topham C, Strunck J (2021). Dupilumab-associated ocular surface disease: clinical characteristics, treatment, and follow-up. Cornea.

[19] Mima Y, Tsutsumi E, Ohtsuka T, Ebato I, Nakata Y, Kubota T, Norimatsu Y (2024). A case of refractory vernal keratoconjunctivitis showing improvement after the administration of upadacitinib for the treatment of atopic dermatitis. Diagnostics (Basel).

[20] Torres T, Yeung J, Prajapati VH (2025). Real-world effectiveness and safety of dupilumab, tralokinumab, and upadacitinib in patients with atopic dermatitis: a 52-week international, multicenter retrospective cohort study. Dermatol Ther (Heidelb).

